# Urgent endoscopic submucosal dissection of an obstructing, bleeding giant duodenal “Brunneroma”

**DOI:** 10.1016/j.vgie.2025.11.009

**Published:** 2025-11-28

**Authors:** Georgios Kalopitas, Elisabet Maristany Bosch, Alessandro Rimondi, Katie Planche, Marco Gulotta, Alberto Murino, Edward John Despott

**Affiliations:** 1 Royal Free Unit for Endoscopy, The Royal Free Hospital, University College London Institute for Liver and Digestive Health, London, UK; 2 Department of Radiology, Royal Free London NHS Foundation Trust, London, UK

## Abstract

**Background and Aims:**

Endoscopic submucosal dissection (ESD) is an advanced technique for the en bloc resection of complex GI lesions, although its use as an urgent alternative to surgery has rarely been reported. This video demonstrates the urgent application of ESD using the Saline-Immersion/irrigation TEchnique (SITE) to treat a giant, bleeding, and obstructing Brunner’s gland hamartoma.

**Methods:**

A 36-year-old man presented with melena, syncopal episodes, and severe anemia. A 2-month history of postprandial discomfort and recent black stools was reported. Upon presentation, the patient was hypotensive and tachycardic. CT imaging showed a large duodenal filling defect, and the urgent gastroscopy revealed a giant pedunculated lesion at the first part of the duodenum with an ulcerated stalk and visible vessels. Initial hemostasis was achieved, but recurrent bleeding prompted urgent SITE-ESD.

**Results:**

The lesion was successfully resected en bloc in under 40 minutes. Because of the lesion's size, retrieval through the pylorus required snare cutting into smaller pieces, facilitated by SITE. The patient recovered without adverse events and was discharged on day 4. Histopathology confirmed a Brunner’s gland hamartoma without dysplasia.

**Conclusions:**

This case demonstrates that ESD can serve as an urgent, organ-preserving definitive option in specific cases. SITE enhances lesion handling and safe retrieval, especially when size limits conventional removal.

## Introduction

Endoscopic submucosal dissection (ESD) is an advanced technique for resecting en bloc complex GI lesions.^1^ This video demonstrates a case of a patient with a giant bleeding and obstructing duodenal Brunner’s gland hamartoma necessitating an ESD using the Saline Immersion/irrigation TEchnique (SITE).^2^ In this video, another advantage of SITE^2^ is presented because the luminal conduction afforded by SITE was used to effectively snare cut the giant lesion in order to be safely retrieved through the pylorus.

## Case presentation

**Figure 1:**
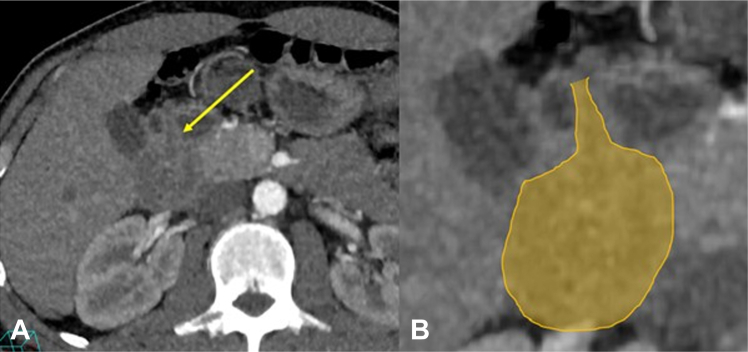
**A,** Arterial phase CT axial image showing a large filling defect distending the duodenum (*arrow*). **B,** Magnified view showing the lesion in *yellow color*.

**Figure 2:**
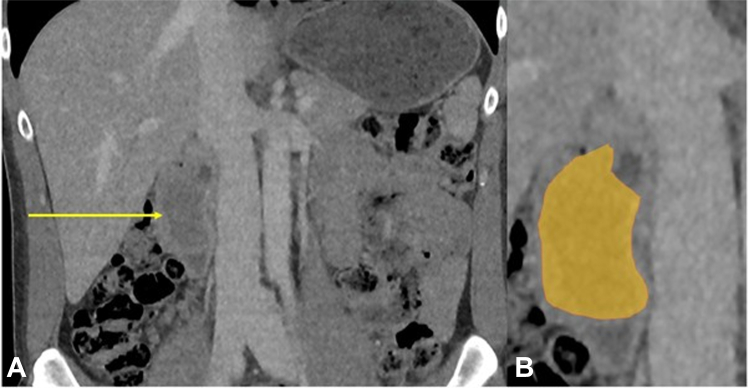
**A,** Coronal portal venous phase CT showing a low-density filling defect in the duodenum (*arrow*). **B,** Magnified view showing the lesion in *yellow color*.

A 36-year-old man presented to the emergency department with melena and syncopal episodes. A 2-month history of gradually worsening postprandial fullness/discomfort and recent black stools was reported. Upon presentation, the patient was hypotensive (blood pressure 97/51 mm Hg), tachycardic (102 bpm), and anemic (hemoglobin [Hb] 8.5 g/dL). Within 6 hours of admission, the patient was transferred to the intensive care unit because of severe ongoing bleeding (Hb dropped to 6.2 g/dL despite transfusion with 2 units of packed red blood cells). An urgent abdominal CT scan revealed a large low-density filling defect distending the duodenum (Figs. 1 and 2).

**Figure 3:**
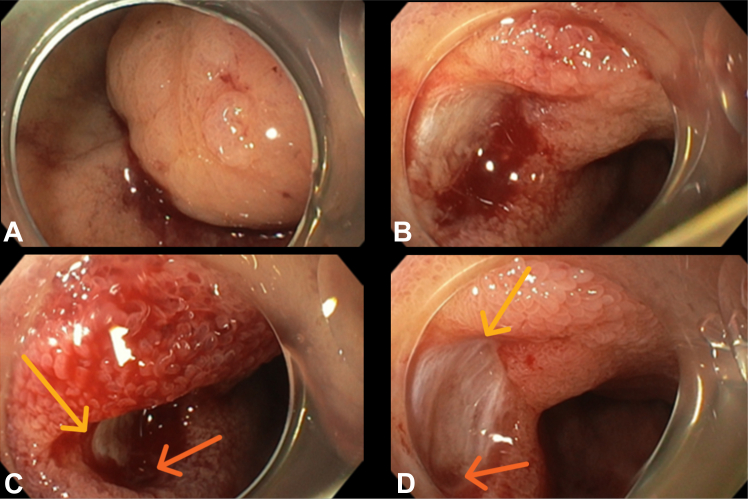
Urgent upper GI endoscopy showing a giant pedunculated lesion (**A**) at the first part of the duodenum, prolapsing into the third part, with an ulcerated stalk (**B**) (*yellow arrow*) and visible vessels (**C and D**) (*orange arrow*).

The urgent upper GI endoscopy showed a giant pedunculated lesion at the first part of the duodenum, prolapsing into the third part, with an ulcerated, bleeding stalk and visible vessels, consistent with the source of hemorrhage. The identified lesion was significantly narrowing the duodenal lumen, raising concern for impending obstruction. Initial hemostasis was applied using 10 mL of adrenaline injection (1:10.000) and 3 mL of protein-matrix gel, PuraStat (3-D Matrix, Tokyo, Japan) (Fig. 3). Despite the initial hemostasis, recurrent bleeding necessitated urgent ESD within 24 hours from the patient's admission.

## Procedure

**Figure 4:**
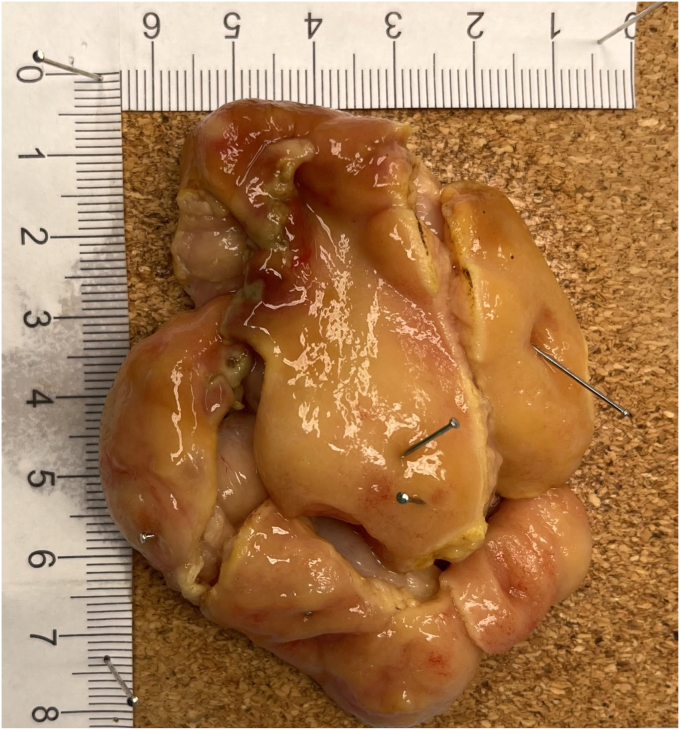
The excised giant duodenal lesion was resected en bloc but had to be cut into smaller pieces for safe retrieval. The resected lesion measured 80 × 60 × 20 mm.

The whole procedure was performed using SITE (Video 1, available online at www.videogie.org). The lesion was initially approached from the anal side on retroflexion, where a mucosal incision was made using a 2.0-mm ball-tipped needle-type knife (FlushKnife BTs 2.0 mm [Fujifilm, Tokyo, Japan]). A scissor-type ESD knife (ClutchCutter 3.5 mm [Fujifilm]) was used for the highly vascular areas of the 30-mm semipedicle, where, given the bleeding presentation and ulcerated appearance of the stalk, a large feeding vessel was suspected. Careful dissection with selective precoagulation of visible vessels was performed under direct visualization. The giant lesion was successfully resected en bloc in <40 minutes. However, because of its size, lesion retrieval through the pylorus was not feasible because the lesion was too big to be delivered through the pylorus. The giant lesion got trapped within the Roth Net (STERIS, Dublin, Ireland), which had to be cut with high-power argon plasma coagulation. SITE facilitated snare cutting of the lesion into smaller pieces, enabling its safe retrieval (Fig. 4). The resected lesion measured 80 × 60 × 20 mm.

## Outcome

**Figure 5:**
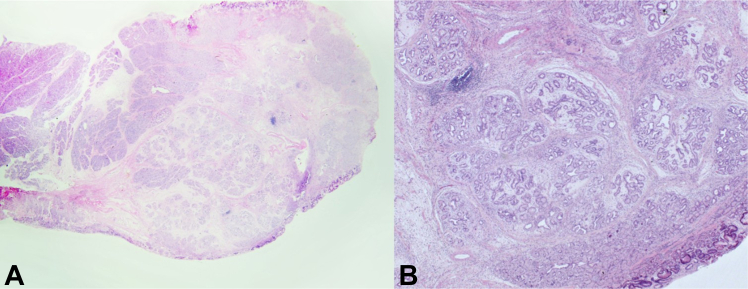
Hematoxylin and eosin–stained sections of the resected lesion. **A,** Low magnification (approximately ×2) showing lobulated Brunner’s-type glands with patchy inflammation. **B,** Medium magnification (approximately ×10) demonstrating preserved glandular architecture without dysplasia. The lesion was diagnosed as a Brunner’s gland hamartoma.

**Figure 6:**
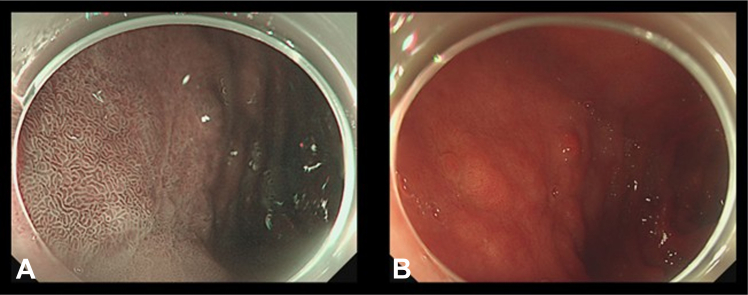
Six-month follow-up endoscopy without any evidence of recurrence (**A and B**).

The patient's recovery was uneventful, and the patient was discharged without any adverse events 4 days later. Histopathology showed a Brunner’s gland hamartoma with no dysplasia (Fig. 5). On follow-up 6 months after resection (Fig. 6), endoscopy demonstrated complete mucosal healing with no evidence of recurrence. The patient remained asymptomatic throughout a 12-month follow-up period, during which his Hb normalized to 13.4 g/dL and remained stable, indicating durable clinical success.

## Conclusions

In conclusion, ESD can serve as an effective, urgent, definitive treatment in select cases, offering an organ-preserving alternative to major abdominal surgery. In this case, ESD was chosen over other resection techniques because it provided direct visualization of the dissection plane, enabling precise control of recurrent bleeding and safe resection of a giant lesion with an ulcerated stalk and anatomic configuration suggestive of impending obstruction. This allowed for timely intervention before surgical management became unavoidable. Unlike underwater ESD, which uses nonconductive water that dilutes surface electrolytes and reduces diathermy efficiency, SITE uses continuous isotonic saline irrigation solution, providing an electrically conductive medium that enhances knife performance and reliable coagulation. In addition, for giant lesions that are resected en bloc but cannot be retrieved intact, the luminal conduction afforded by SITE facilitates effective snare cutting, enabling safe extraction through narrow anatomical confines such as the pylorus. Although SITE offers important advantages, it should be applied with caution in emergent or anatomically complex scenarios because significant bleeding may require temporary suctioning and gas insufflation to localize the source.

## Patient Consent

The patient in this article has given written informed consent to publication of their case details.

## Declaration of Generative Artificial Intelligence and Artificial Intelligence–assisted Technologies in the Writing Process

Generative artificial intelligence and artificial intelligence–assisted technologies were not used for the preparation of this work by the authors.

## Disclosure

The following authors disclosed financial relationships: E.J. Despott has received educational grants in support of conference organization, and honoraria from Fujifilm, Pentax, Olympus, and Ambu. A. Murino has received speaker honoraria from Olympus Medical, Laborie, Boston Scientific, and Fujifilm Healthcare Europe. The other authors disclosed no financial relationships.

## Supplementary data

Video 1 Urgent endoscopic submucosal dissection of the giant duodenal Brunneroma using the Saline-Immersion/irrigation TEchnique
